# The Evaluation of the Periodontal Status of Hemodialysis Patients with End-Stage Renal Disease

**DOI:** 10.3390/jcm11040975

**Published:** 2022-02-13

**Authors:** Elżbieta Dembowska, Aleksandra Jaroń, Joanna Rasławska-Socha, Ewa Gabrysz-Trybek, Joanna Bladowska, Szymon Gacek, Grzegorz Trybek

**Affiliations:** 1 Departament of Periodontology, Pomeranian Medical University, 70-111 Szczecin, Poland; elzbieta.dembowska@pum.edu.pl (E.D.); joanna.raslawska@pum.edu.pl (J.R.-S.); szgacek@gmail.com (S.G.); 2 Department of Oral Surgery, Pomeranian Medical University, 70-111 Szczecin, Poland; jaronola@gmail.com; 3 Department of Diagnostic Imaging and Interventional Radiology, Pomeranian Medical University, 71-242 Szczecin, Poland; ewa_gabrysz@wp.pl; 4 Department of General and Interventional Radiology and Neuroradiology, Wroclaw Medical University, 50-369 Wrocław, Poland; joanna.bladowska@umed.wroc.pl

**Keywords:** chronic kidney disease, periodontitis, periodontal status, dialysis, hemodialysis, oral hygiene, CKD, end-stage renal disease

## Abstract

Chronic kidney disease (CKD) is an increasingly common condition observed in developing countries. Similarly, a high prevalence of gingivitis and periodontitis is observed. There are reports in the literature about the interrelationship between chronic kidney disease and periodontitis pathophysiology. This dissertation attempts to: assess the extent of gingivitis and periodontitis in a group of patients with the end-stage renal disease treated with hemodialysis compared to healthy subjects. The study included 200 subjects: 100 hemodialysis patients (HD) and 100 healthy control subjects (K). Periodontal status was assessed by measuring pocket depth (PD) clinical level of connective tissue attachment (CAL). Gingival inflammation indices Gingival Index (GI) and Bleeding on Probing (BOP) were also performed. PD with a depth of more than 6mm was found in 25% of the HD group and 5% of the K group. CAL ≥ 5 mm was found in 55% of HD and 24% of the K group. As defined by Page and Eke, severe periodontitis was found in 21% of HD, and 4% of K. Moderate gingivitis was noted in 55% of HD and 5% of the K group. The mean values of the BOP index in the HD group were 32.08% and in the K group 3.09%. The HD group had a higher incidence and severity of gingivitis and periodontitis than the control group.

## 1. Introduction

Today, chronic kidney disease (CKD) is one of the significant public health problems due to the systematically increasing number of patients, especially patients with end-stage renal disease [1,2]. In recent years, a worldwide increase in the prevalence of chronic kidney disease has been observed [3]. The majority of hypertension and diabetes, one of CKD’s most critical etiologic factors, should be emphasized [4]. On the other hand, patients with chronic kidney disease are at high risk of developing cardiovascular diseases such as coronary artery disease, myocardial infarction, and cardiac failure [5,6]. The mortality rate in hemodialysis patients is estimated at 20% per year, more than half of which are deaths due to the aforementioned cardiovascular diseases [7]. In the early stages of chronic kidney disease, no increased incidence of periodontal or dental disorders has been found [8]. Only pathophysiological phenomena accompanying chronic kidney disease in the last stage result in changes in the oral cavity. Due to the complex morphology of this region, the changes may affect not only the teeth and periodontium but also the oral mucosa. In end-stage chronic obstructive pulmonary disease, the senses of taste and smell may also be impaired, resulting in malnutrition and weight loss [9,10]. Salivary gland dysfunction may cause dryness of the oral mucosa and result in difficulties with food intake [9,10,11,12]. Low zinc levels, a cofactor of gustin observed in serum, saliva, and leukocytes, increase the taste sensitivity threshold [13]. There is also a correlation between high urea concentrations and dysfunction of the sense of taste [10]. Uremic patients are often diagnosed with halitosis in the form of uremic odor from the mouth [14]. Periopathogens such as *Porphyromonas gingivalis*, *Tanarella forsythia*, *Prevotella intermedia*, and *Aggregatibacter actinomycetemcommitans* are more frequent in hemodialysis patients. Candida albicans were also more prevalent in saliva in hemodialysis patients than in controls [15]. The oral mucosa of patients with CDK, as a result of complex mechanisms of water–electrolyte imbalance, constant irritation with toxic metabolites present in saliva, and a frequent tendency to xerostomia, become more susceptible to the occurrence of disorders and pathological changes [9]. One of the characteristic features of patients with MS is the propensity for oral mucosal infections and inflammation within the periodontal tissues. Abnormalities in calcium–phosphate metabolism often lead to abnormalities observed in bone tissue and dental morphology [16]. However, there have been reports in the literature about the insignificant effect of diagnosed secondary hyperparathyroidism in hemodialysis patients on periodontal status and alveolar levels [17]. Furthermore, in patients on peritoneal dialysis, a higher incidence of periodontitis and an association of periodontitis with poorer patient nutrition and elevated values of inflammatory parameters in blood serum has been observed [18]. Moreover, the results of numerous studies have shown that the values of indices determining periodontal inflammation: Gingival Index (GI) according to Löe and Silness and Papilla Bleeding Index (PBI) according to Saxer and Mühlemann in dialysis patients reached higher values than in the control group of healthy subjects [19,20]. Yoshihara et al. [21] observed a correlation between renal dysfunction and bone metabolism and a higher percentage of study sites with Clinical Attachment Level (CAL) of ≥6 mm.

The COVID-19 epidemic has significantly impacted the incidence, diagnosis, and treatment of kidney disease, including end-stage renal failure. Impaired access to specialized treatment may have had a significant impact on the outcome of the underlying disease and associated diseases such as periodontitis [22,23].

The aim of our study was to evaluate the severity of gingivitis and periodontitis in patients with the end-stage renal disease treated with hemodialysis compared to healthy subjects.

## 2. Materials and Methods

The study was conducted in a group of hemodialyzed patients with chronic kidney disease, in cooperation with three dialysis stations and the Department of Periodontology at the Medical University. The Bioethics Committee of the Medical University approved the study (Resolution No. K0012/45/11). Individuals qualified for the study gave informed consent to participate and were informed in detail about its purpose and course.

The study comprised 200 subjects, including group HD (*n* = 100)—100 hemodialyzed subjects with end-stage renal disease, both genders, aged 19 to 85 years—and group K (*n* = 100)—100 subjects without chronic kidney disease, aged 18 to 83 years, adequate in sex and age distribution to the study group, constituting the control group.

The inclusion criteria for the study group (HD) were: duration of dialysis for a minimum of three months, end-stage form of chronic renal failure, and informed consent to participate in the study. The exclusion criteria were: taking immunosuppressive or cytotoxic drugs currently or in the past; disseminated malignancy; antibiotic therapy at the time of the study or within the last three months; and acute infectious disease in the oral cavity, pharynx, and salivary glands at the time of the study (Table 1).

The criterion for inclusion in the control group (K) was the adequacy concerning sex and age to the distribution of patients of the study group (HD) and informed consent to participate in the study.

### 2.1. Physical Examination

The general medical history included information on general health, duration of dialysis therapy, and concomitant diseases.

### 2.2. Evaluation of the Oral Cavity

The examination was performed using a dental mirror and a Hu Frieda UNC15 Periodontal Screening Probe scaled in 1 mm increments. The measurement was made at well-defined points at each tooth in the oral cavity. According to the WHO guidelines for oral health examinations, probe placement under the nail plate was used to obtain visible anemia of the skin in this area [24]. The force is described as 20–25 N.

### 2.3. Clinical Evaluation of the Periodontal Status

Detailed periodontal status was determined on physical examination in the form of Probing Depth (PD), which is the distance between the gingival margin and the bottom of the pocket, and Clinical Attachment Level (CAL), which is the measurement of the distance from the enamel–cement border to the bottom of the pocket, measured in millimeters. PD was measured using the Hu Friedy Screening Probe UNC15 periodontal probe at six measurement points at each tooth on the buccal and lingual sides distally, medially, and mesially, respectively. Periodontal health was determined using the Gingival Index (GI) according to Löe and Silness [25]. Bleeding on Probing (BOP) symptoms, according to Ainamo and Bay [26], were also observed to describe the extent of periodontal inflammation in patients. To qualify the periodontal condition, the periodontitis classification according to Page and Eke [27] was used, taking into account the obtained PD and CAL measurements at interproximal sites.

### 2.4. Statistical Analysis

The Kolmogorov–Smirnov test was used to determine the normality of the distribution of the variables. Characterization of variables was performed using means, standard deviations, and outliers. Student’s *t*-test and Mann–Whitney test were used to examine the differences between the study groups (HD, K). Pearson’s test and Fisher’s exact test were used to reflecting relationships between discontinuous variables. Analysis of variance (ANOVA) or Kruskal–Wallis test was also used to describe the groups. Using frequency and number of occurrences, discontinuous variables were described, between which relationships were characterized using Pearson’s χ^2^ (chi-square) test. Spearman’s rank correlation was referred to in evaluating the correlation between discontinuous variables (nominal and ordinal) and continuous variables, illustrated by the correlation coefficient r and probability p. Statistically significant differences were represented by a confidence level of *p* < 0.05. A confidence level value within the range of *p* = 0.051–0.099 was interpreted as a trend, not strictly statistically significant. Statistical analysis was performed using STATA 11 program license number 30110532736.

## 3. Results

### 3.1. Characteristics of the Studied Groups of Patients

The study included 200 subjects, including 100 hemodialysis patients with end-stage renal disease, of both sexes, aged 19 to 85 years, and 100 patients without chronic kidney disease, aged 18 to 83 years, adequate in terms of sex and age distribution to the study group, constituting the control group. Among hemodialysis patients, the mean age was 55 years (±16.43), of which 42% (*n* = 42) were female, and 58% (*n* = 58) were male. The control group, which reported a mean age of 52 years (±15.46), consisted of 43% (*n* = 43) women and 57% (*n* = 57) men.

During statistical analysis of mean age in both study groups—study group (HD) and control group (K)—no statistically significant differences were found (*p* = 0.77). The control group, concerning age and gender, was matched to the study group among patients attending the Periodontology Outpatient Clinic of the Medical University for diagnostic and dental treatment. An analysis of the distribution of groups concerning gender was performed. The number of men in both groups (HD, K) totaled 57.5% (*n* = 115), while the number of women concerning the total study population was 42.5% (85), respectively. There were no statistically significant differences in gender distribution in both groups (HD, K) of subjects (*p* = 0.87). Statistical analysis of age distribution in both groups (HD, K) showed no significant differences (*p* = 0.52843).

The following general medical conditions were found to be present in patients of the hemodialysis (HD) group: hypertension in 72% (*n* = 72), diabetes in 29% (*n* = 29), coronary artery disease in 14% (*n* = 14), osteoporosis in 11% (*n* = 11), glomerulonephritis in 9% (*n* = 9), rheumatoid arthritis (RA) in 9% (*n* = 9), endocrine disorders (does not include hyperparathyroidism) in 5% (*n* = 5), psychiatric diseases in 6% (*n* = 6), hyperparathyroidism in 4% (*n* = 4), other autoimmune diseases (e.g., systemic lupus, SLE) in 2% (*n* = 2), genetic disorders (e.g., polycystic kidney disease) in 2% (*n* = 2), viral hepatitis in 4% (*n* = 4), drug toxicity in 1% (*n* = 1) and drug addiction in 1% (*n* = 1) of cases.

### 3.2. Oral and Periodontal Status in the Study Groups

The PD values obtained in both groups (HD, K) were as follows: PD depth up to 3 mm was noted in 48% (*n* = 48) of subjects in the control group (K) and 29% (*n* = 29) of issues in the study group (HD); PD between 4 and 6 mm was noted in 47% (*n* = 47) of the control group (K) and 46% (*n* = 46) of the study group (HD). Periodontal pockets greater than 6 mm were found in 5% (*n* = 5) of patients in the control group and 25% (*n* = 25) of patients in the study group (HD). Statistically significant differences were found in the distribution of periodontal pocket depth values in both study groups (*p* < 0.01) (Table 2).

The mean values of the PD measurements were also evaluated and are shown in Table 3. The following summary also includes pocket depths at the interproximal sites. The mean value of periodontal pocket depths at all the studied sites was recorded as 2.8 mm (±1.18) in the study group (HD) compared to the mean PD of 1.93 mm (±0.48) in the control group. It should be noted that the differences in the parameters described showed a statistically significant difference (*p* = 0.000). In the HD group, the mean PD at interproximal sites was equal to 3.14 (±1.49). In the K group, the value was 2.08 (±0.52). The differences presented are statistically significant (*p* = 0.0000).

In the context of Page and Eke classification [27], the number of interproximal sites with PD values ≥ 5 mm was determined. Statistically significant differences between the HD and K groups were shown in the number of periodontal pockets PD ≥ 5 mm at interproximal sites (*p* < 0.001). There was a mean of 6.63 (±13.44) pockets in the HD group and 1.17 (±3.79) pockets in the K group.

Statistical analysis of CAL measurements was performed similarly (Table 4). CAL values in the 1–2 mm range were observed in 42% (*n* = 42) of subjects in the control group (K) and 40% (*n* = 40) of the study group (HD). CAL values between 3 and 4 mm were observed in 34% (*n* = 34) of subjects in the control group (K) and 5% (*n* = 5) of subjects in the study group (HD). CAL equal to or exceeding 5 mm was noted in 55% (*n* = 55) of subjects in the study group (HD) and 24% (*n* = 24) of subjects in the control group (K). Statistical comparison of CAL values in both groups showed differences with a significant level of significance (*p* < 0.0001).

Table 5 compares the mean values of CAL measurements with a special note on interproximal sites. The mean CAL value at all sites examined was 1.73 mm (±1.86) in the HD group and 0.39 mm (±0.85) in the K group. These differences show statistical significance (*p* = 0.001). CAL measurement values at interproximal sites were analyzed. The interproximal sites were characterized by a mean CAL value of 1.93 mm (±2.15) in the HD group and 0.42 (±0.92) in the K group. The differences described are statistically significant (*p* = 0.001). The mean number of interproximal sites with CAL values ≥ 4 mm and CAL values ≥ 6 mm was described. For the HD group, the data are as follows: CAL mean 21.79 (±22.64) interproximal sites with CAL ≥ 4 mm and mean 4.92 (±11.61) interproximal sites with CAL ≥ 6 mm; in the K group, a mean of 5.58 (±12.45) interproximal sites with CAL ≥ 4 mm and 1.09 (±4.32) interproximal sites with CAL ≥ 6 mm. These differences are statistically significant (*p* < 0.01) (Table 5).

The diagnosis of periodontitis in the evaluated groups (HD, K) referred to the definition of periodontitis according to Page and Eke [27], taking into account CAL and PD values at interproximal sites. According to the data in Table 6, no or mild periodontitis was diagnosed in 37% (*n* = 37) of the study group (HD) and 67% (*n* = 67) of the control group (K). In contrast, moderate periodontitis was diagnosed in 42% (*n* = 42) of the HD group and 29% (*n* = 29) of the K group study participants. Severe, advanced periodontitis was observed in as many as 21% (*n* = 21) of subjects in the HD group and in 4% (*n* = 4) of subjects in the K group. The differences between the evaluated groups showed high statistical significance (*p* < 0.0001).

As shown in Table 7, higher GI values, according to Löe and Silness, were more common in the study group (HD) compared to the control group (K). GI values between 2.1 and 3.0 were observed in 7% (*n* = 7) of subjects in the HD group and none in the K group. GI values between 1.1 and 2.0 were observed in 55% (*n* = 55) of subjects in the HD group and 5% (*n* = 5) of subjects in the K group. In contrast, values of 0.1–1.0 were noted in 38% of the HD group and 60% (*n* = 60) of the K group. In the HD group, no GI values were found in the range <0.1, which is represented by 35% (*n* = 35) of the subjects in the K group. The described differences between the studied groups are characterized by high statistical significance (*p* = 0.000).

High statistical significance was also demonstrated when comparing the mean GI values in the studied groups (HD, K) (*p* = 0.000), presented in Table 8. The mean GI value was 1.23 (±0.56) in the HD group and 0.32 (±0.35) in the K group.

Table 9 illustrates that the BOP index also shows higher mean values in the study group (HD) than the control group (K). These differences are statistically significant (*p* = 0.000).

The relationships between the gender of the subjects and the mean values of periodontal status and oral hygiene parameters were also analyzed, as summarized in Table 10. No statistically significant differences were found between the mean PD and CAL and indices: BOP and PI in the study groups (*p* > 0.05). Only the GI index showed a statistically significant difference concerning gender in the K group (*p* = 0.046). No analogous relationship was described in the study group (HD). According to the data included in Table 10, there were no statistically significant differences between the assessed parameters of periodontal status and oral hygiene in the context of the gender of the subjects.

## 4. Discussion

In the present study, the periodontal status is expressed by PD and CAL parameters. In our study, the mean PD value was recorded at 2.81 (±1.18) mm in the study group (HD) and 1.93 (±0.48) mm in the control group (K). The differences described showed a statistically significant relationship (*p* = 0.000). In the classification of periodontitis proposed by the American Academy of Periodontology (AAP), periodontal pocket (PD) depths greater than 6 mm are associated with the diagnosis of advanced disease [28,29]. In the present study, PD with depths greater than 6 mm was found in 25% of hemodialysis patients and 5% of control subjects. These differences are statistically significant (*p* < 0.01).

Studies by Chhokra et al. [30] and Frankenthal et al. [17] obtained similar mean PD values to those presented in the present study: 2.82 (±0.49) mm and 2.92 (±0.14) mm, respectively. Higher mean PD values were observed in the studies of Cholewa et al. [31] and Jenabian et al. [32] and were 3.45 (±0.80) mm and 4.41 (±1.4) mm, respectively.

Smaller periodontal pocket depths were observed in the studies by Bayraktar et al. [33], Cengiz et al. [34], Kadiroglu et al. [35], and Marakoglu et al. [36]. Bayraktar et al. [37] compared the PD periodontal pocket depths in hemodialysis (HD) (PD 1.88 ± 0.40 mm), peritoneal dialysis (DO) (PD 1.89 ±0.52 mm), and control (K) groups of healthy subjects (PD 1.94 ± 0.62 mm). The authors showed no statistically significant differences and relatively low mean PD values in these study groups compared to their study. The differences in the obtained results may be explained by the different ages of the patients in the HD group [37]. For example, in the study by Bayraktar et al. [33], the mean age was: 46 ± 15 years in the HD group, 44 ± 12 years in the DO group, and 46 ± 18 years in the K group; in our study, the mean age was: 55 (±16.43) years in the HD group and 52 (±15.46) years in the K group [33]. Similarly, in the study by Kadiroglu et al. [35], the mean PD values were lower than in the author’s analysis, being 1.93 (±0.44) mm and 1.72 (±0.46) mm in the hemodialysis group with high and low CRP levels, respectively. The mean age of the patients in this study was 43.1 (±13.1) and 39.2 (±14.6) years, and this group was also younger than the authors’ study. Marakolgu et al. [36] also observed lower mean PD values of 1.8 (±0.6) mm. This study included a group of 36 hemodialysis patients also with a lower mean age of 50.4 (±14.2) years. Cengiz et al. [34] reported mean PD values of 2.3 (±0.6) mm, respectively.

In the AAP classification of periodontitis, CAL values above 4 mm are significant for advanced forms of the disease [28,29]. In the authors’ study, CAL above 4 mm was observed in as many as 55% of hemodialysis patients, compared to 24% of control subjects. These differences show a great level of significance. There were also statistically significant differences in mean CAL values: in hemodialyzed patients, they were equal to 1.73 mm (±1.86), and in the control group, they were 0.39 mm (±0.85) (*p* = 0.001).

In the study of Marinho et al. [20], clinical attachment loss was found to be more frequent (96.2%) in the group of patients with chronic kidney disease, including those on dialysis, compared to the control group (47.4%). The described relationships were highly statistically significant (*p* < 0.000). Moreover, the authors [20] found clinical attachment loss in 100% of hemodialysis patients participating in the study. In a survey by Ajithkrishnan et al. [38], clinical attachment loss in the range of 4–5 mm was observed

In a study by Ajithkrishnan et al. [38], clinical attachment loss in the range of 4–5 mm was observed in 36.18% of hemodialysis patients and only 4.61% of patients in the control group. Thorman et al. [39], in a study of a group of 93 patients with varying degrees of chronic kidney disease, found more significant clinical attachment loss in the hemodialysis patient group than in peritoneal dialysis patients. Thorman et al. [20] described mean CAL values in hemodialysis patients of 2.2 mm. The authors noted that loss of connective tissue attachment is more frequently observed in the group with end-stage CDK.

Similarly, Gonçalves et al. [40] found a frequent clinical attachment loss and periodontitis in hemodialysis patients (47.1%). Gonçalves et al. [40] reported mean CAL values of 2.30 (±0.96) mm. The minimum CAL values were 1.37 mm, and the maximum values were −5.27 mm. Frankenthal et al. [17] reported relatively high values of attachment loss in their study. They averaged 4.43 (±0.29) mm in 35 hemodialysis patients with secondary hyperparathyroidism with a mean age of 43.77 (±2.36) years. Similarly, Jenabian et al. [32] found CAL values averaging 3.98 (±1.61) mm.

The results’ differences can be explained by the heterogeneous research methodology used, for example, due to differences in the size and age spread of the patient groups studied. Furthermore, the method of periodontal measurements may affect the results obtained; for example, in the study of Frankenthal et al., data [17] measurements were made within the Ramfjörd index teeth, which may have affected the data obtained. The literature [19] highlights the influence of ethnic, racial, socioeconomic, and behavioral contexts on the findings. Papers by authors from different cultural, socioeconomic, and geographic backgrounds may present different prevalences of periodontal pathology.

Moreover, the care organization for hemodialysis patients varies according to region, economic factors, and health care organizations in different countries. Borawski et al. [19] described their findings as representative of the northeastern Polish region and neighboring geographic regions, including Belarus, Lithuania, and the western parts of Ukraine and Russia. Similarly, studies by Turkish authors [33,34,35,36,41] present data that are characteristic of the Middle East region.

Based on the literature analysis and our studies, we demonstrated that periodontitis is very common in hemodialyzed patients and is characterized by a more advanced presentation. Periodontitis was also frequent much earlier, before diagnostics in the group of hemodialyzed patients. The literature suggested the need for further studies on periodontal status in hemodialysis patients and those with chronic kidney disease [19]. It is also pointed out that the issue of periodontitis may often be overlooked in the care of hemodialysis patients. In the present study, the periodontitis classification, according to Page and Eke [27], which considers the obtained PD and CAL measurements, was used to qualify the periodontal status. This is a sensitive, modern classification that considers interproximal sites, the most indicative for periodontitis progression [42].

Numerous scientific reports place Page and Eke’s definition as the “gold standard” for periodontitis diagnosis. Zawada et al. [42] draw attention to one of the most important features of this definition, which determines its high sensitivity, is the periodontal condition on the interdental surfaces of the teeth. The omission of vestibular surfaces reduces the likelihood of hyperdiagnosis due to the prevalence of recession in this exact location [42].

In the authors’ study, we observed a higher prevalence of periodontitis in hemodialysis patients compared to healthy subjects. Concerning Page and Eke’s definition, up to 21% of hemodialysis subjects described severe periodontitis, compared to 4% of control subjects. Similarly, 42% of hemodialysis subjects and 29% of control subjects were diagnosed with mild to moderate periodontitis. Healthy periodontium was found in 37% of hemodialysis patients and 67% control subjects.

Similarly, in a study by Gonçalves et al. [40], clinical attachment loss and periodontitis were frequent in a group of hemodialysis patients, with the authors reporting periodontitis in 47.1% of the subjects. In the study by Nadeem et al. [43], periodontitis was observed in as many as 57.5% of hemodialysis patients. Parkar et al. [38] also reported a higher prevalence of periodontitis and poor oral hygiene in hemodialysis patients.

It should also be pointed out that some studies did not report statistically significant differences between the periodontal status of hemodialysis patients and the control group.

In the study by Marakoglu et al. [36], none of the hemodialysis patients studied had clinically healthy periodontium without signs of inflammation. The authors [36] noted the presence of intermediate gingivitis in 44% of hemodialysis (HD) patients, mild gingivitis in 17% of HD, and advanced gingivitis in 14% of HD patients. Some studies have observed a relationship between dialysis time and oral and periodontal status [34].

Postbiotics or lactoferrin in oral surgery or periodontology to support the treatment of periodontal disease, among others, is increasingly discussed in the literature [44,45]. These are mostly natural substances, which may support the treatment of periodontal diseases to the same extent as chlorhexidine. Their use as an adjunct to SRP in patients with end-stage renal failure may have many benefits. This topic, in our opinion, is worth further exploration.

A current topic widely discussed in the literature is COVID-19, its impact on all aspects of patients’ lives, including oral health. In addition to the lack of a specific treatment regimen for COVID-19, new treatment regimens contain various drugs that may affect the oral, including periodontal, condition. In addition, the pandemic that may have caused difficult access to specialized treatment may affect the overall health of patients with end-stage renal disease, including their periodontal status. Unfortunately, the study in this publication was conducted before the COVID-19 pandemic, so these data were not included, which is a limitation of the study.

## 5. Conclusions

There was a higher incidence and severity of gingivitis and periodontitis in hemodialysis patients compared to controls. Advanced periodontitis is up to five times more frequent in hemodialysis patients compared to the control group. Regular clinical follow-up of both the underlying disease and associated diseases, such as periodontitis, may prevent deterioration of patients’ overall health. The use of additional adjunctive measures to treat periodontal disease may improve the health status of the subjects.

## Figures and Tables

**Table 1:** Eligibility criteria for the study group (HD).

Study Group Inclusion Criteria:	Study Exclusion Criteria:
Duration of dialysis for a minimum of 3 months	Taking immunosuppressive or cytotoxic drugs now or in the past
Presence of end-stage chronic renal failure	Disseminated malignancy
Informed consent to participate in the study	Antibiotic therapy at the time of the study or within less than 3 months at the time of the study
	Acute infectious disease of the mouth, throat, or salivary glands at the time of the study
	Active viral infections at the time of the study and in the time preceding the study

**Table 2:** Comparison of the distribution of periodontal pocket depths (PD) in both study groups (HD, K).

Depth of Periodontal Pockets (PD) (mm)	Study Group (HD)		Control Group (K)		Total
≤3 mm	29	29.00%	48	48.00%	77
4–6 mm	46	46.00%	47	47.00%	93
>6 mm	25	25.00%	5	5.00%	30
Total	100		100		200
Pearson’s χ^2^	df = 2		13.79		*p* < 0.01
Spearman’s R rank	t = 36597		0.25		*p* < 0.01

**Table 3:** Mean periodontal pocket (PD) depths and mean number of PD interproximal pockets ≥ 5 mm in the study groups (HD, K).

Pocket Depth Values		$\bar{x}$	±SD	Min.	Max.	Q25	Me	Q75	*p*
Periodontal pocket depths (PD) Depths of pockets at all study sites (mm)	Study group (HD)	2.81	1.18	0.92	7.27	2.04	2.75	3.34	0.000
	Control group (K)	1.93	0.48	0.58	3.64	1.69	1.87	2.16	
Mean periodontal pocket depths at interproximal sites (mm)	Study group (HD)	3.14	1.49	1.01	9.50	2.20	2.97	3.70	0.000
	Control group (K)	2.08	0.52	0.58	4.20	1.81	2.02	2.28	
Number of interproximal pockets ≥ 5 mm	Study group (HD)	6.63	13.44	0.00	62.00	0.00	1.00	4.50	0.000
	Control group (K)	1.17	3.79	0.00	24.00	0.00	0.00	0.00	
Depths of periodontal pockets in the maxilla (mm)	Study group (HD)	2.81	1.31	0.92	7.08	1.90	2.59	3.23	0.000
	Control group (K)	1.98	0.48	0.58	3.64	1.71	1.90	2.24	
Depths of periodontal pockets in the mandible (mm)	Study group (HD)	2.79	1.14	0.81	7.31	2.05	2.68	3.35	0.000
	Control group (K)	1.89	0.53	0.58	4.16	1.62	1.83	2.11	

**Table 4:** Comparison of the distribution of clinical values of the level of connective tissue attachment (CAL) in both study groups (HD, K).

CAL (mm)	Study Group (HD)		Control Group (K)		Total
1–2 mm	40	40.00%	42	42.00%	82
3–4 mm	5	5.00%	34	34.00%	39
≥5 mm	55	55.00%	24	24.00%	79
Total	100		100		200
Pearson’s χ^2^	33.78		df = 2		*p* < 0.001
Spearman’s R rank	0.18		t = 26028		*p* < 0.01

**Table 5:** Mean clinical attachment level (CAL) values in the study groups.

CAL		$\bar{x}$	±SD	Min.	Max.	Q25	Me	Q75	*p*
CAL loss at all tested sites (mm)	Study group (HD)	1.73	1.86	0.00	7.30	0.00	1.46	2.89	0.0001
	Control group (K)	0.39	0.85	0.00	4.88	0.00	0.06	0.33	
CAL loss at interproximal sites (mm)	Study group (HD)	1.93	2.15	0.00	9.41	0.00	1.67	3.14	0.0001
	Control group (K)	0.42	0.92	0.00	5.21	0.00	0.05	0.36	
Number of all tested sites with CAL loss > 5 mm	Study group (HD)	12.26	18.92	0.00	80.00	0.00	4.00	15.50	0.0000
	Control group (K)	2.96	9.07	0.00	54.00	0.00	0.00	1.00	
Number of interproximal sites with loss ≥ 4 mm	Study group (HD)	21.79	22.64	0.00	73.00	0.00	18.50	36.50	0.0000
	Control group (K)	5.58	12.45	0.00	68.00	0.00	1.00	5.00	
Number of interproximal sites with CAL loss ≥ 6 mm	Study group (HD)	4.92	11.61	0.00	58.00	0.00	0.00	3.00	00024
	Control group (K)	1.09	4.32	0.00	29.00	0.00	0.00	0.00	

**Table 6:** Diagnosis of periodontitis according to Page and Eke [27] in the study groups (HD, K).

Page and Eke Classification	Study Group (HD)		Control Group (K)		Total
No or slight inflammation	37	37%	67	67%	104
Moderate inflammation	42	42%	29	29%	71
Severe inflammation	21	21%	4	4%	25
Total	100		100		200
Pearson’s χ^2^	22.59		df = 2		*p* < 0.001
Spearman’s R rank	−0.33		t = −4928		*p* < 0.001

**Table 7:** Frequency of gingivitis according to Gingival Index (GI) by Löe and Silness in the examined groups (HD, K).

Mean Gingival Index (GI) Values	Study Group (HD)		Control Group (K)		Total
<0.1	0	0%	35	35%	35
0.1–1.0	38	38%	60	60%	98
1.1–2.0	55	55%	5	5%	60
2.1–3.0	7	7%	0	0%	7
Total	100		100		200
Pearson’s χ^2^	94.08		df = 2		*p* = 0.000
Spearman’s R rank	−0.68		t = −13.21		*p* = 0.000

**Table 8:** Mean Gingival Index (GI) values according to Löe and Silness in the study groups.

Gingival Index (GI) Values According to Löe and Silness		$\bar{x}$	±SD	Min.	Max.	Q25	Me	Q75	*p*
Gingival Index (GI) values	Study group (HD)	1.23	0.56	0.15	3.00	0.72	1.24	1.51	0.000
	Control group (K)	0.32	0.35	0.00	1.42	0.05	0.14	0.52	
Gingival Index (GI) values for the maxilla	Study group (HD)	1.12	0.59	0.05	3.00	0.60	1.11	1.45	0.000
	Control group (K)	0.27	0.36	0.00	1.83	0.00	0.08	0.50	
Gingival Index (GI) values for the mandible	Study group (HD)	1.28	0.55	0.15	3.00	0.94	1.28	1.54	0.000
	Control group (K)	0.35	0.37	0.00	1.47	0.06	0.20	0.65	

**Table 9:** Mean values of BOP index in the study groups.

Mean Values of Bleeding on Probing (BOP)		$\bar{x}$	±SD	Min.	Max.	Q25	Me	Q75	*p*
Average BOP values	Study group (HD)	32.08	27.45	0.00	100.00	10.36	26.04	46.67	0.000
	Control group (K)	3.09	7.18	0.00	50.00	0.00	0.00	2.86	
Mean values of (BOP) for the maxilla	Study group (HD)	26.06	26.63	0.00	100.00	5.36	18.75	39.06	0.000
	Control group (K)	3.44	12.11	0.00	100.00	0.00	0.00	0.00	
Average values of index (BOP) for mandible	Study group (HD)	33.91	27.49	0.00	100.00	10.71	28.13	50.00	0.000
	Control group (K)	2.88	5.55	0.00	25.00	0.00	0.00	3.57	

**Table 10:** Mean values of periodontal pocket depth (PD), clinical attachment level (CAL), Gingival Index (GI) according to Löe and Silness, and Plaque Index (PI) in the study groups concerning gender.

Issue			*n*	$\bar{x}$	±SD	Q25	Me	Q75	*p*	r^2^	r
Periodontal pocket depths (PD) (mm)	Control group (K)	male	57	1.99	0.41	1.73	1.93	2.20	0.1755	0.02	0.14
		female	43	1.86	0.54	1.61	1.78	2.03			
	Study group (HD)	male	58	2.71	1.14	1.97	2.58	3.33	0.2980	0.01	0.11
		female	42	2.96	1.23	2.07	2.86	3.44			
Clinical attachment level (CAL) (mm)	Control group (K)	male	57	0.46	0.97	0.00	0.08	0.52	0.3410	0.01	0.10
		female	43	0.30	0.65	0.00	0.05	0.29			
	Study group (HD)	male	58	1.58	1.77	0.00	0.96	2.85	0.3214	0.01	0.10
		female	42	1.95	1.98	0.00	1.77	3.13			
Gingival Index (GI) according to Löe and Silness	Control group (K)	male	57	0.37	0.37	0.07	0.27	0.61	0.0713	0.03	0.18
		female	43	0.25	0.31	0.02	0.12	0.39			
	Study group (HD)	male	58	1.23	0.52	0.72	1.17	1.52	0.9899	0.00	0.00
		female	42	1.23	0.61	0.90	1.29	1.50			
Plaque Index (PI)	Control group (K)	male	0.53	0.43	0.17	0.43	0.81	0.53	0.4363	0.01	0.08
		female	0.47	0.39	0.09	0.37	0.81	0.47			
	Study group (HD)	male	1.91	0.63	1.53	1.97	2.44	1.91	0.9320	0.00	0.01
		female	1.92	0.73	1.30	2.00	2.45	1.92			

## Data Availability

Data available on request.

## References

[1] Hallan I.S., Stevens P. (2010). Screening for chronic kidney disease: Which strategy?. J. Nephrol..

[2] Levey A.S., Atkins R., Coresh J., Cohen E., Collins A., Eckardt K., Nahas M.E., Jaber B.L., Jadoul M., Levin A. (2007). Chronic kidney disease as a global public health problem: Approaches and initiatives—A position statement from Kidney Disease Improving Global Outcomes. Kidney Int..

[3] Schiepatti A., Remuzzi G. (2005). Chronic renal diseases as a public health problem: Epidemiology, social, and economic implications. Kidney Int..

[4] Chan M., Ostermann C. (2013). Outcomes of Chronic Dialysis Patients in the Intensive Care Unit. Crit. Care Res. Pract..

[5] Tuegel C., Bansal N. (2017). Heart failure in patients with kidney disease. Heart.

[6] Shoji T., Abe T., Matsuo H., Egusa G., Yamasaki Y., Kashihara N., Shirai K., Kashiwaji A. (2012). Chronic Kidney Disease, Dyslipidemia and Atherosclerosis. J. Atheroscler. Thromb..

[7] Go A.S., Chertow G.M., Fan D., McCulloch C.E., Hsu C.Y. (2004). Chronic Kidney Disease and the Risks of Death, Cardiovascular Events, and Hospitalization. N. Engl. J. Med..

[8] Garcez J., Limeres Posse J., Carmona I., Feijoo J.F., Dios P.D. (2009). Oral health status of patients with a mild decrease in glomerular filtration rate. Oral Surg. Oral Med. Oral Pathol. Oral Radiol. Endod..

[9] Kho H., Lee S., Chung S., Kim Y. (1999). Oral manifestations and salivary flow rate, pH, and buffer capacity in patients with end-stage renal disease undergoing hemodialysis. Oral Surg. Oral Med. Oral Pathol. Oral Radiol. Endod..

[10] Korytowska A., Szmeja Z. (1993). Zachowanie się węchu i smaku u chorych z przewlekłą niewydolnością nerek leczonych hemodializą. Otolar. Pol..

[11] Sokołowska-Trelka A., Grzebieluch W., Dubiński B. (2005). Problemy stomatologiczne u chorych na schyłkową niewydolność nerek. Dent. Med. Probl..

[12] Wilczyńska-Borawska M., Borawski J., Stokowska W. (2004). Czynniki ryzyka utraty zębówu pacjentów przewlekle hemodializowanych. Dent. Med. Probl..

[13] Sudesh K., Prasad A., Parviz R. (1982). Zinc deficiency: A reversible complication of uremia. Am. J. Clin. Nutr..

[14] Keles M., Tozoglu U., Uyanik A., Eltas A., Bayindir Y., Cetinkaya R., Bilge O.M. (2011). Does peritoneal dialysis affect halitosis in patients with end-stage renal disease?. Perit. Dial. Int..

[15] Castillo A., Mesa F., Liebana J., Garcia-Martinez O., Ruiz S., Garcia-Valdecasas J., O’Valle F. (2007). Periodontal and oral microbiological status of an adult population undergoing haemodialysis: A cross-sectional study. Oral Dis..

[16] Proctor R., Kumar N., Stein A., Moles D. (2005). Oral and dental apects of chronic renal failure. J. Dent. Res..

[17] Frankenthal S., Nakhoul S., Machtei E., Greek E., Ardekian L., Laufer D., Peled M. (2002). The effect of secondary hyperparathyroidism and hemodialysis therapy on alveolar bone and periodontium. J. Clin. Periodontol..

[18] Cengiz M., Bal S., Gokcay S., Cengiz K. (2007). Does periodontal disease reflect atherosclerosis in continuous ambulatory peritoneal dialysis patients?. J. Periodontol..

[19] Borawski J., Wilczyńska-Borawska M., Stokowska W., Myśliwiec M. (2007). The periodontal status of pre-dialysis chronic kidney disease and maintenance dialysis patients. Nephrol. Dial. Transplant..

[20] Sobrado Marinho J.S., Tomás Carmona I., Loureiro A., Limeres Posse J., García Caballero L., Diz Dios P. (2007). Oral health status in patients with moderate-severe and terminal renal failure. Oral Med. Oral Patol. Oral Chir. Bucal..

[21] Yoshihara A., Deguchi T., Nobuhiro H., Miyazaki H. (2007). Renal function and periodontal disease in elderly Japanese. J. Periodontol..

[22] Nshimyiryo A., Barnhart D.A., Cubaka V.K., Dusengimana J.M.V., Dusabeyezu S., Ndagijimana D., Umutesi G., Shyirambere C., Karema N., Mubiligi J.M. (2021). Barriers and coping mechanisms to accessing healthcare during the COVID-19 lockdown: A cross-sectional survey among patients with chronic diseases in rural Rwanda. BMC Public Health.

[23] McAdams M., Ostrosky-Frid M., Rajora N., Hedayati S.S. (2021). Effect of COVID-19 on Kidney Disease Incidence and Management. Kidney360.

[24] World Health Organization (2013). Oral Health Surveys: Basic Methods.

[25] Löe H., Silness J. (1963). Periodontal disease in pregnancy. Prevalence and severity. Acta Odontol. Scand..

[26] Ainamo J., Bay I. (1975). Problems and proposals for recording gingivitis and plaque. Int. Dent. J..

[27] Page R.C., Eke P.I. (2007). Case Definitions for Use in Population-Based Surveillance of Periodontitis. J. Periodontol..

[28] American Academy of Periodontology (2000). Parameter on chronic periodontitis with advanced loss of periodontal support. J. Periodontol..

[29] American Academy of Periodontology (2000). Parameter on chronic periodontitis with slight to moderate loss of periodontal support. J. Periodontol..

[30] Chhokra M., Manocha S., Dodward V., Gupta U., Vaish S. (2013). Establishing an Association between Renal Failure and Periodontal Health: A Cross Sectional Study. J. Clin. Diagn. Res..

[31] Cholewa M., Ignasiak W., Radwan-Oczko M. (2013). Stan przyzębia a wskaźnik BMI u chorych dializowanych—Badania pilotażowe. Dent. Med. Probl..

[32] Jenabian N., Mirsaeed A., Ehsani H., Kiakojori A. (2013). Periodontal status of patient’s underwent hemodialysis therapy. Caspian J. Intern. Med..

[33] Bayraktar G., Kurtulus I., Kazancioglu R., Bayramgurler I., Cintan S., Bozfakioglu S., Besler M., Trablus S., Issever H., Yildiz A. (2008). Evaluation of periodontal parameters in patients undergoing peritoneal dialysis or hemodialysis. Oral Dis..

[34] Cengiz M., Sumer P., Cengiz S., Yavuz U. (2009). The effect of the duration of the dialysis in hemodialysis patients on dental and periodontal findings. Oral Dis..

[35] Kadiroglu K., Kadiroglu E., Sit D., Dag A., Yilmaz E. (2006). Periodontitis is an important and occult source of inflammation in hemodialysis patients. Blood Purif..

[36] Marakoglu I., Gursoy U.K., Demirer S., Sezer H. (2003). Periodontal status of chronic renal failure patients receiving hemodialysis. Yonsei Med. J..

[37] Bots C.P., Poorterman J.H.G., Brand H., Kalsbeek H., Van Amerongen B.M., Veerman E., Nieuw Amerongen A.V. (2006). The oral health status of dentate patients with chronic renal failure undergoing dialysis therapy. Oral Dis..

[38] Parkar S.M., Ajithkrishnan C.G. (2012). Periodontal status in patients undergoing hemodialysis. Indian J. Nephrol..

[39] Thorman R., Neovius M., Hylander B. (2009). Clinical findings in oral health during progression of chronic kidney disease to end-stage renal disease in a Swedish population. Scand. J. Urol. Nephrol..

[40] Gonçalves E., Lima D., Albuquerque S., Carvalho J., Cariri T., De Oliveira C. (2011). Avaliação da perda de inserção dentária em pacientes com doença renal crônica em hemodiálise. J. Bras. Nefrol..

[41] Bayraktar G., Kurtulus I., Duraduryan A., Cintan S., Kazancioglu R., Yildiz A., Bural C., Bozfakioglu S., Besler M., Trablus S. (2007). Dental and periodontal findings in hemodialysis patients. Oral Dis..

[42] Zawada Ł., Chrzęszczyk D., Konopka T. (2012). Definicje zapaleń przyzębia w wybranej grupie dorosłych mieszkańców Wrocławia. Dent. Med. Probl..

[43] Nadeem M., Stephen L., Schubert C., Davids M. (2009). Association between periodontitis in end-stage renal disease. S. Afr. Dent. J..

[44] Butera A., Gallo S., Pascadopoli M., Taccardi D., Scribante A. (2022). Home Oral Care of Periodontal Patients Using Antimicrobial Gel with Postbiotics, Lactoferrin, and Aloe Barbadensis Leaf Juice Powder vs. Conventional Chlorhexidine Gel: A Split-Mouth Randomized Clinical Trial. Antibiotics.

[45] Trybek G., Jedliński M., Jaroń A., Preuss O., Mazur M., Grzywacz A. (2020). Impact of lactoferrin on bone regenerative processes and its possible implementation in oral surgery—A systematic review of novel studies with metanalysis and metaregression. BMC Oral Health.

